# Structure-activity relationships of steroid and sterol neuromodulators on inflammatory markers in a murine microglial cell line

**DOI:** 10.1016/j.jsbmb.2026.106969

**Published:** 2026-02-27

**Authors:** Hong-Jin Shu, Douglas F. Covey, Charles F. Zorumski, Steven Mennerick

**Affiliations:** a Department of Psychiatry, Washington University School of Medicine, St Louis, MO 63110, USA; b Department of Developmental Biology, Washington University School of Medicine, St Louis, MO 63110, USA; c Taylor Family Institute for Innovative Psychiatric Research, Washington University School of Medicine, St Louis, MO 63110, USA

**Keywords:** Neuroactive steroids, oxysterols, cytokines, anti-inflammatory, GABA_A_ receptor, structure-activity

## Abstract

Neuroactive steroids are allosteric modulators of GABA_A_ receptors and are implicated in the etiology and treatment of neuropsychiatric disorders. Existing treatments are helpful but have drawbacks. Neuroactive steroids recently gained attention as rapidly acting antidepressants in postpartum depression and other indications. Unlike other GABA_A_ receptor modulators, neurosteroids may possess anti-inflammatory actions, potentially contributing to therapeutic benefit. Here we seek to understand neuroactive steroid structure-activity relationships relevant to these anti-inflammatory effects. We used murine microglial BV2 cells challenged with lipopolysaccharide (LPS) as an inflammation model. We investigated structure-activity profile of neuroactive steroids and oxysterol-like compounds on cytokine transcription. LPS increased transcripts for cytokines IL-1β, IL-6 and TNF-α. Both allopregnanolone and its enantiomer significantly suppressed these LPS-induced increases, with no effect in the absence of LPS.

**Conclusions:**

Our results suggest that neuroactive steroids exhibit a distinct structure-activity profile compared with their GABA_A_ receptor modulation effects. Certain neuroactive steroids may selectively target neuro-inflammation. The enantiomer of AlloP could be a tool compound to differentiate anti-inflammatory effects of neuroactive steroids from GABAergic and other enantioselective effects.

## 1. Introduction

Neuroinflammation is implicated in a variety of neurodegenerative and neuropsychiatric disorders, including major depressive disorder [1-6]. Although inflammation is a normal mechanism that protects the brain from protein aggregates, neuronal injury, and infection [1], the intensity and duration of neuroinflammation determine whether inflammation is beneficial or detrimental. Some forms of inflammation are clearly harmful, and early intervention provides the best treatment outcomes. For instance, depression is one of the most common psychiatric disorders, affecting humans throughout their lifespan [7,8]. Although disentangling correlation and causation is challenging [9,10], recent evidence suggests a causative role for inflammation [11,12]. Chemical stimulation of the immune system mimics symptoms of depression [13,14]. Here, in the context of tests of varied steroid-like structures, we elucidate neuroactive steroids as a newer class of pharmacological neuroinflammatory interventions with relevance to neuropsychiatry.

Several classes of steroids and sterols have neuromodulatory roles at transmitter receptors and have therapeutic potential in neuropsychiatric indications. Neurosteroids and synthetic analogues have recently captured attention as rapidly acting antidepressants [15]. Brexanolone (allopregnanolone, AlloP) was the first FDA-approved medicine to treat postpartum depression [16-18] and may have broader benefit in major depressive disorder and seizure disorders [19,20]. Although their best-known action is that of positive modulation of GABA_A_ receptors [21,22], AlloP inhibits systemic inflammatory pathways, including inhibition of toll-like receptor (TLR4 and TLR7) responses [23]. Oxysterols are another emerging potential treatment for neuropsychiatric indications [24,25]. The abundant natural oxysterol 24S-hydroxycholesterol has positive modulatory effects at NMDAR-type glutamate receptors [26]. 25-hydroxycholesterol is microglial derived and has complex immune effects in animals [27-29] and also interacts with NMDA receptors [30].

Structure-activity relationships for these neuromodulators have been explored for neurotransmitter receptors but not for inflammation. A key observation is that the unnatural enantiomer of AlloP is much weaker than AlloP at GABA_A_ receptors [31,32]. Further, stereochemistry of the hydroxyl group at carbon 3 of AlloP is critical, but stereochemistry of the reduction at carbon 5 is not [32]. Given that AlloP and related compounds can influence inflammatory signaling, it is important to clarify which structural features of neurosteroids confer anti-inflammatory activity and whether those features align with GABA_A_ receptor activity.

To address structure-activity considerations for inflammation, we used LPS-challenged BV2 murine microglial-derived cells [33-35] as a screening platform to compare the effects of AlloP, steroisomeric and enantiomeric compounds, other neurosteroids analogues, and selected oxysterol analogues on pro-inflammatory cytokine expression. We aimed to identify compounds that suppress LPS-induced cytokine transcription, to assess concentration-dependence, exclude cytotoxicity, and finally to determine whether anti-inflammatory activity correlates with GABA_A_ receptor modulatory profiles.

Our results are consistent with a different structure-activity profile for neuroactive steroids than that exhibited for GABA_A_ receptor modulation. Thus, our results suggest that only a restricted number of neuroactive steroids engage neuroinflammation, potentially limited to the naturally occurring neurosteroid AlloP and its enantiomer. The unnatural enantiomer could be a novel tool compound to separate anti-inflammatory effects of neuroactive steroids from GABAergic and other enantioselective effects.

## 2. Materials and methods

### 2.1. Reagents

Lipopolysaccharide (LPS) from *Escherichia coli* O111:B4 (L2630, Lot 028M4021V) and dexamethasone (DEX) (D4902) were purchased from Millipore Sigma (St. Louis, MO). Neuroactive steroids, AlloP and its enantiomer (*ent*-AlloP), progesterone and *ent*-progesterone, etiocholanolone (Etio) and *ent*-Etio, and KK-129 were prepared by the Covey laboratory. 3β5αP, pregnenolone sulfate (PS) and 25-hydroxycholesterol (25-HC) were purchased from Millipore Sigma. Oxysterol variants of 24S-hydroxycholesterol (24S-HC), SGE-201 and SGE-301were synthesized by previously published methods [26] and were gifts from Sage Therapeutics (Boston, MA).

### 2.2. Cell culture of BV2 cells and treatment

The BV2 murine microglial-derived cell line was maintained at 37°C with 5% CO_2_ in a humidified incubator. Cells were cultured with high-glucose DMEM medium (Gibco, Thermo-Fisher Scientific) supplemented with 10% fetal bovine serum and 1% penicillin streptomycin. Cells were plated in 12-well plates a day before experiments at a density of 3.75 × 10^5^/ml. At 70% confluence, LPS (100 ng/ml) was applied to each well for overnight treatment, with or without the neuroactive steroids co-applied. Controls utilized the DMSO vehicle. In addition to naïve cells, the neurosteroid analogues alone (without LPS) or DMSO alone were used as comparison conditions.

### 2.3. Total RNA extraction and cDNA synthesis in BV2 microglia cells

Total RNA was isolated from the cell lysates using the RNeasy Mini Kit (QIAGEN GmbH, Germany) in accordance with the manufacture’s protocols. RNA was quantified using a ND1000 nanodrop spectrophotometer. cDNA was synthesized from 1 μg of RNA using a SuperScript II Reverse Transcriptase (Invitrogen, Thermo-Fisher Scientific) using random primers.

### 2.4. Quantitative real-time polymerase chain reaction (qPCR)

The gene expression levels of IL-1β, IL-6 and TNF-α in BV2 cells were determined by real-time quantitative PCR (qPCR). cDNA was mixed with Master Mix Universal for SYBR Green (Lamda Biotech, St. Louis, MO) and primers specific to the genes of interest. Real-time qPCR amplification was performed on QuantStudio 3 (Applied Biosystems, Foster City, CA).

Primers: For IL-1β, the forward sequence was *Fw: GGCAACTGTTCCTGAACTCAACTG* and reverse was *Rv: CCATTGAGGTGGAGAGCTTTCAGC*. For IL-6, *Fw: CCACTTCACAAGTCGGAGGCTT* and *Rv: CCAGCTTATCTGTTAGGAGA*. For TNFα, *Fw: CCTATGTCTCAGCCTCTTCT* and *Rv: CCTGGTATGAGATAGCAAAT*. For IL-10, *Fw: ATAACTGCACCCACTTCCCA* and *Rv: GGGCATCACTTCTACCAGGT*. For GAPDH, *Fw: AGGTCGGTGTGAACGGATTTG and Rv: TGTAGACCATGTAGTTGAGGTCA.*

The threshold cycle (Ct) value was defined as the cycle number at which the fluorescence crossed a fixed threshold above the baseline. For relative quantification, fold changes were measured using the ΔΔCt method. For each sample, the Ct value of cytokine mRNA was compared with the GAPDH endogenous control as ΔCt (ΔCt = Ct_cytokine_ – Ct_GAPDH_). The cytokine mRNA fold change in experimental sample relative to control samples was determined using 2^−ΔΔCt^ (ΔΔCt = ΔCt_experiment_ – Δct_control_).

### 2.5. Solvent controls and compound concentration-response curve

Control conditions in main Figures correspond to DMSO solvent controls (0.1% maximum). There was no effect of DMSO on IL-6 transcription compared to the LPS alone group (1.04 ± 0.11, N = 8, Supplemental Figure 1 A).

AlloP and *ent*-AlloP were evaluated at a fuller range of concentrations than other compounds. BV2 cells were co-treated with compounds at concentrations of 0.03, 0.1, 0.3 and 1 μM during overnight LPS administration. Total RNA was isolated as described in Section 2.3 and assayed by qPCR as in Section 2.4.

### 2.6. Measurement of cytokine levels in BV2 culture medium

Cytokine protein levels in BV2 cell culture medium were determined by Luminex FLEXMAP 3D technology (Millipore Sigma, St. Louis). A predesigned MillPlex of 32 mouse cytokine was used. A group of culture medium samples was selected with their matched qPCR sample dataset. Cytokine concentrations were expressed as pg/ml in medium and subjected to further correlation analysis. The full original files of the MilliPlex results are uploaded to FigShare (https://doi.org/10.6084/m9.figshare.30053995.v1).

### 2.7. Cell viability assay

For cell viability assays, BV2 cells were seeded onto 12-well plates one day prior to the experiment. AlloP, *ent*-AlloP, Etio and *ent*-Etio, representing steroids with effects on cytokine expression, were administered with and without LPS at a concentration of 10 μM overnight. Cell viability was assessed using the Trypan Blue method (Sigma Life Science), following the manufacturer’s protocol. After incubation, cells were washed with PBS, resuspended, and incubated with dye. Cell counts were performed using a hemocytometer. The percentage of dead cells relative to the total cell population was calculated to determine cell viability. Representative brightfield images were acquired using a Nikon Eclipse microscope with a 20x objective.

### 2.8. Statistical analysis

Statistical analyses were performed using GraphPad Prism version 10.4.0 software. Power analyses were used on pilot datasets to generate target N values. Data are presented as the mean ± SEM. The differences in cytokine levels between LPS alone and compound-treated groups were tested by the Wilcoxon signed-rank test. Multiple corrections were not performed because experiments were performed largely independently of each other and are grouped in figures for ease of description only. Exact *P* values are displayed in figures. Sample sizes are indicated by data points on graphs. Comparisons between protein and RNA expression levels were compared by linear regression, detailed in the figure legend.

## 3. Results

### 3.1. AlloP and ent-AlloP inhibit LPS-induced transcription of pro-inflammatory cytokines IL-1β, IL-6 and TNF-α

**Fig. 1.**
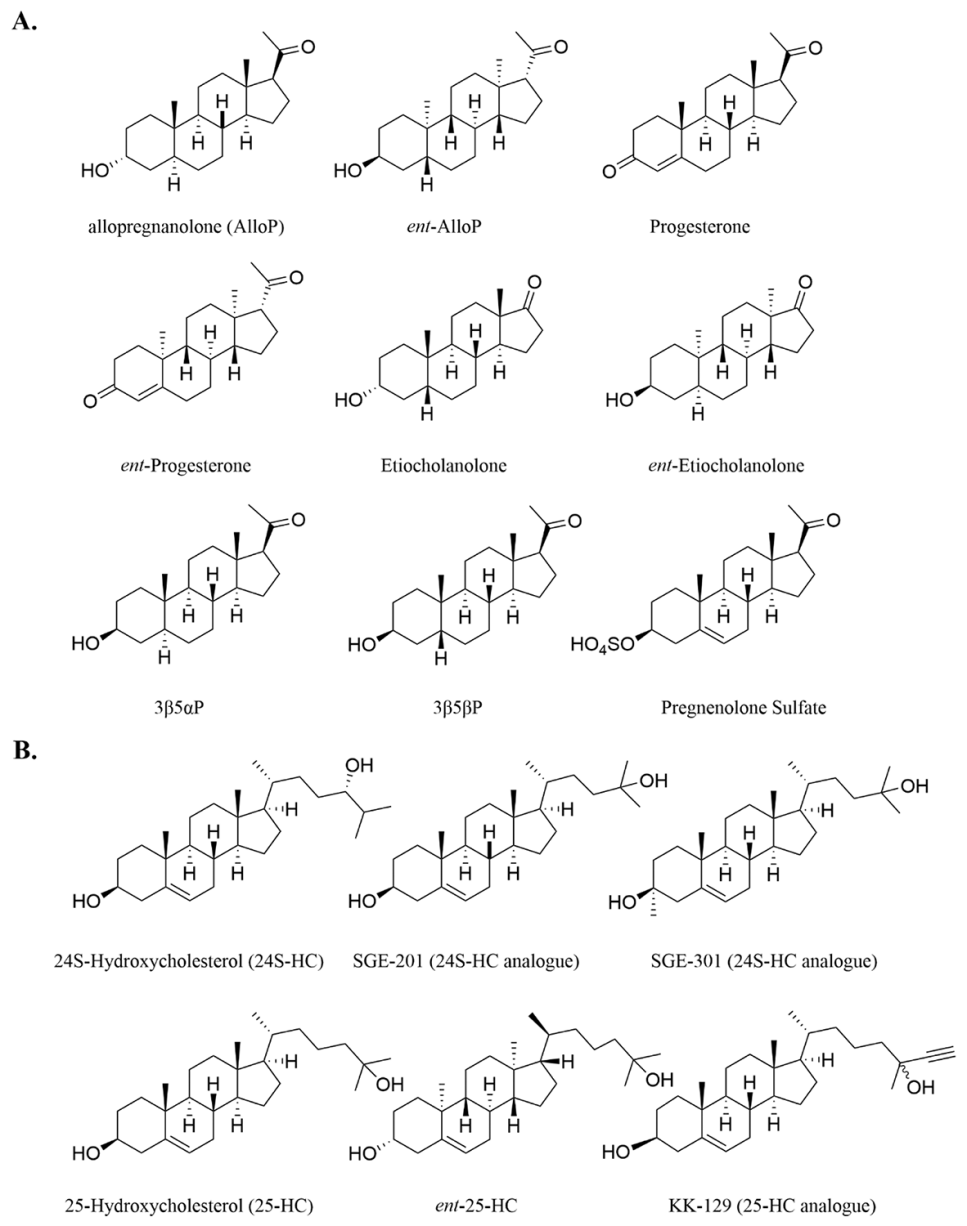
Chemical structures of neuroactive steroids and oxysterols used in the experiments. A. Neuroactive steroids and their enantiomers. **B.** Side-chain oxysterols.

The chemical structures of compounds selected for the present studies are illustrated in Fig. 1. These include natural neurosteroids, their unnatural enantiomers, oxysterols, and oxysterol analogues. We prioritized AlloP, since it is in clinical use and is also a canonical naturally occurring neurosteroid. A priority comparator was the unnatural enantiomer of AlloP (*ent*-AlloP), which exhibits weak GABA_A_ receptor activity [31]. Oxysterols are oxidative cholesterol metabolites with various signaling roles distinct from neurosteroids [24].

**Fig. 2.**
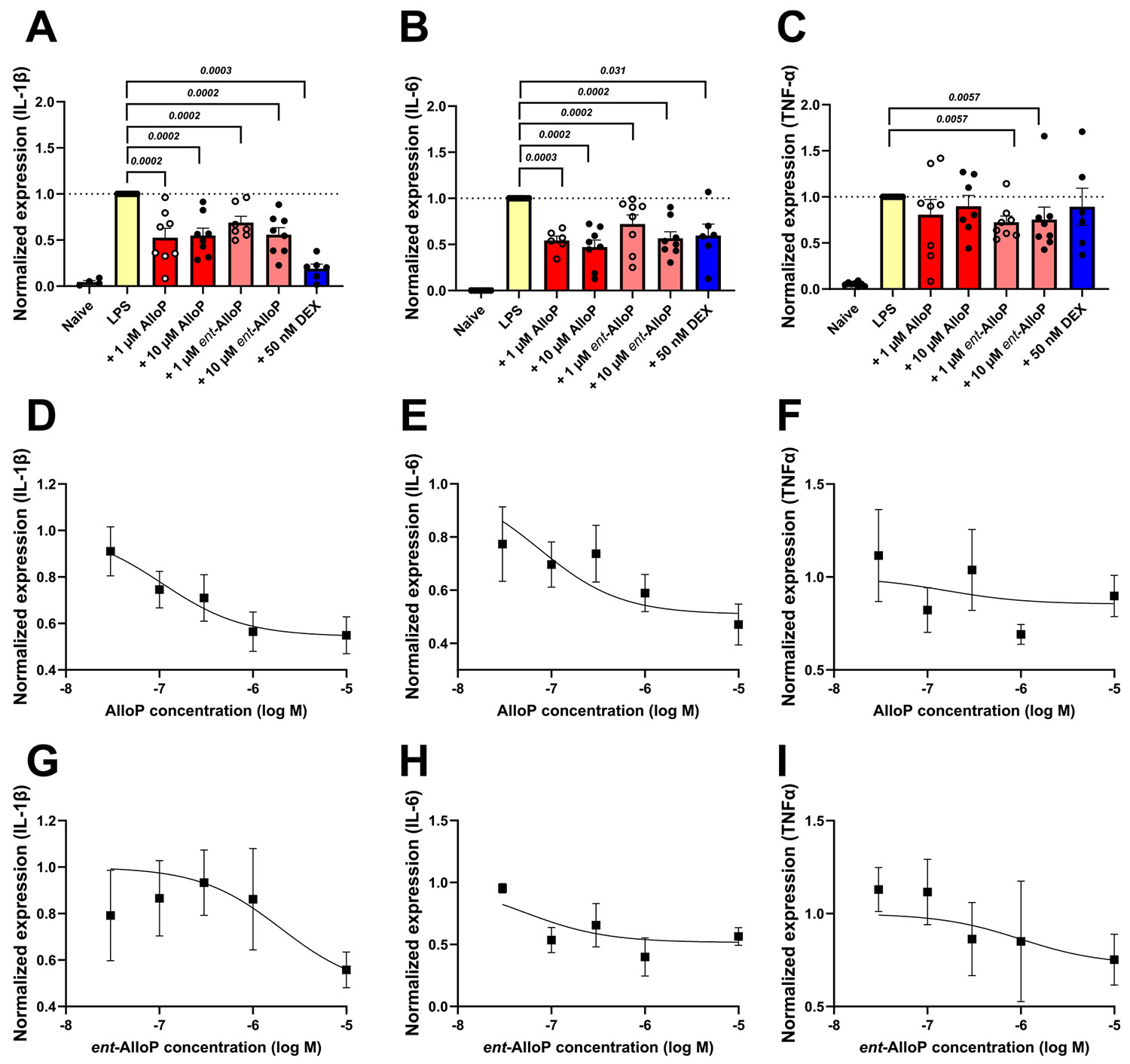
Anti-inflammatory effect of AlloP and its enantiomer *ent*-AlloP in murine microglia (BV2) cultures challenged by LPS administration using real-time quantitative PCR. LPS was used at 100 ng/ml for 16–18 h. AlloP and *ent*-AlloP were co-applied with LPS or were applied in the absence of LPS (Supplemental Figure 1). Dexamethasone (DEX) (blue bar) served as a comparison. Dose-dependence was investigated using two concentrations of neuroactive steroid (1 μM shown as empty circles, and 10 μM shown as solid circles). Cells lysates were used for total RNA isolation followed with qPCR assay. A. AlloP and *ent*-AlloP suppress the mRNA expression of the inflammatory cytokine interleukin 1β (IL-1β). AlloP (red bars) and *ent*-AlloP (pink bars) significantly inhibited LPS-induced interleukin 1β expression. B and C: Both AlloP and *ent*-AlloP inhibit the mRNA expression of the inflammatory cytokine interleukin 6 (IL-6) (B) with marginal effects on tumor necrosis factor α (TNF-α) (C). Statistical analyses were performed by the Wilcoxon signed-rank test, and the *P* values are indicated above bars. D-I. The dose-response relationships of AlloP and *ent*-AlloP on cytokine expression in LPS treated BV2 cells were examined using concentrations ranging from 0.03 μM to 10 μM. We observed weak concentration dependence of neuroactive steroid treatment in the context of LPS-induced inflammation, represented as 1.0. Fits are shown and yielded IC50’s < 10 μM and maximum effect asymptotes > 0.5. Data represent N = 3 independent platings.

To investigate the potential effects of neuroactive steroids under neuroinflammatory conditions, we challenged microglial-derived BV2 cells with LPS administration to model cellular inflammation. LPS increased transcription of inflammatory cytokines relative to naïve cells (Fig. 2A-C, yellow bars). The prototypical neurosteroid AlloP, co-applied overnight with LPS, reduced the LPS-induced transcriptional changes of IL1-β by 48% compared to LPS alone at 1 μM. Tenfold higher concentration (10 μM) produced a similar effect (Fig. 2A red bars). The enantiomer of AlloP, *ent*-AlloP is only weakly effective at GABA_A_ receptors but exhibited a similar inhibition of IL-1β mRNA expression at both 1 μM and 10 μM (Fig. 2A pink bars).DEX (50 nM) was used as an anti-inflammatory comparator [36,37]. Overall, AlloP and *ent*-AlloP exhibited effective anti-inflammatory effects when compared to DEX (blue bars in Fig. 2A). The results with IL1-β transcription were largely paralleled with another pro-inflammatory cytokine, IL-6. (Fig. 2B); effects of neuroactive steroids on TNF-α were not as apparent (Fig. 2C). Compounds in the absence of LPS failed to alter cytokine transcription at either 1 μM or 10 μM (Supplemental Figure 1B).

To verify that concentrations of neuroactive steroids chosen represented full effect of steroids, we examined concentrations of 0.03, 0.1, 0.3 and 1 μM for active compounds AlloP and *ent*-AlloP (Fig. 2D-I). We observed weak concentration dependence of neuroactive steroids in the context of LPS-induced inflammation and conclude the effects are near maximum but incomplete at 10 μM.

### 3.2. Structure-activity profile of other steroid structures and their enantiomers on LPS-induced inflammatory responses

**Fig. 3.**
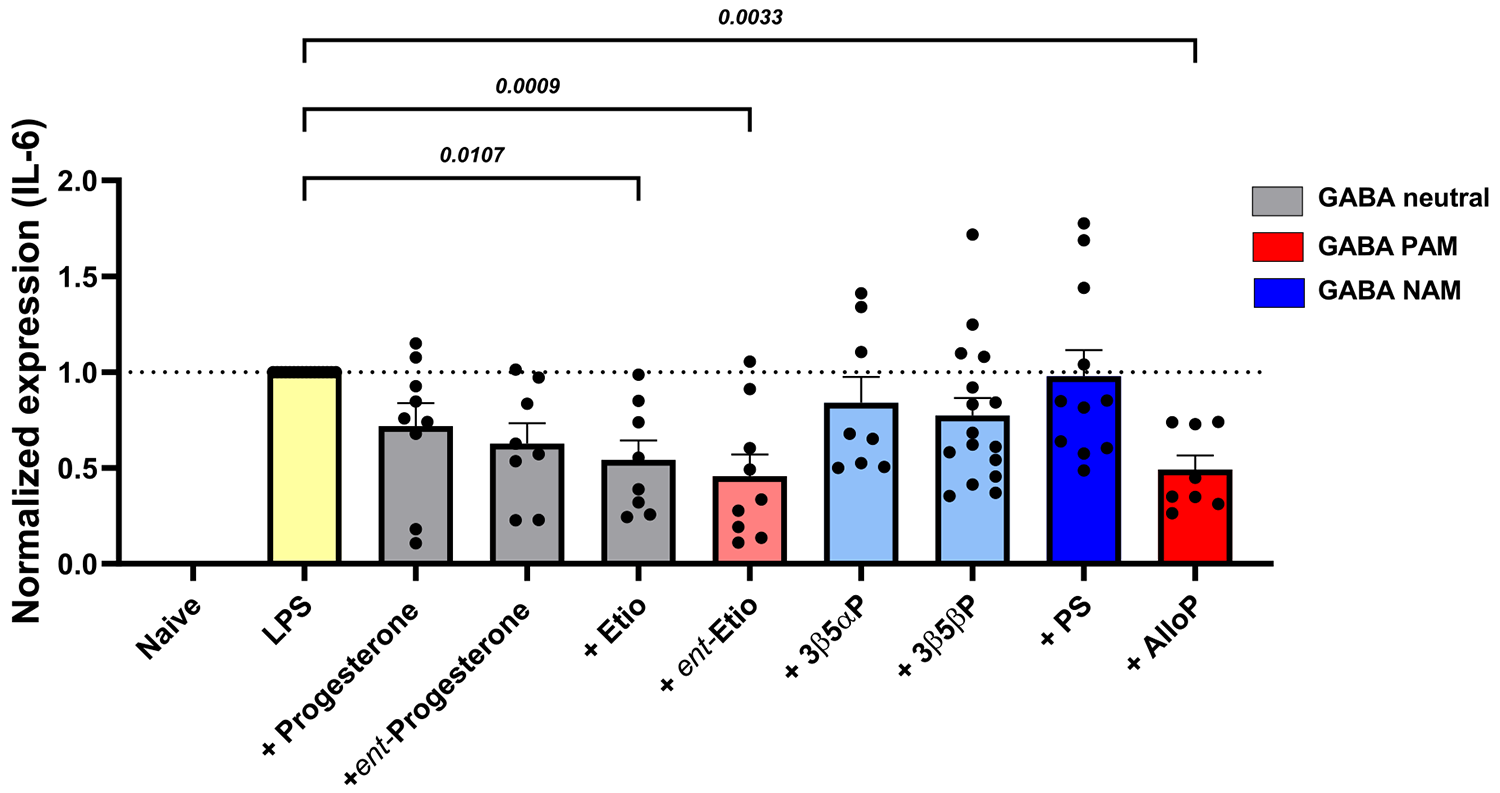
Survey of the anti-inflammation effect (IL-6) across stereoisomers and other analogues. The results of cytokine IL-6 mRNA expression are presented from qPCR results. PAMs of GABA_A_ receptors are shown in red bars, with lighter red color indicating lower GABA PAM potency [21,22], neutral modulators in grey bars, and NAM in blue bars, with lighter blue indicating lower NAM potency [69,70]. All compounds were used at a concentration of 10 μM. Statistical analyses were performed by the ordinary one-way ANOVA test, and the *P* values are indicated above the bars.

To explore the structure-activity relationship of neuroactive steroids for anti-inflammatory effects, we selected a group of compounds representing distinct categories of structures and differing impacts on GABA_A_ receptors (Figs. 1,3, Supplemental Table). Their effects on LPS-induced proinflammatory cytokine transcription were tested at 10 μM using qPCR for IL-6 as a representative cytokine. Fig. 3 shows the effect of steroids with a neutral impact on GABA_A_ receptors (at concentrations < ~5 μM; illustrated in grey bars), including progesterone [38], *ent*-progesterone, and Etio. The three compounds mildly suppressed IL-6 mRNA production. Etio, another neutral modulator of GABA_A_ receptors, and *ent*-Etio a positive modulator of GABA_A_ receptors, [39,40] also both suppressed IL-6 mRNA production (Fig. 3). Pregnenolone sulfate, a negative modulator of GABA_A_ receptors, and 3β5αP, 3β5βP barely impacted IL-6 mRNA expression induced by LPS challenge (Fig. 3 blue bars and light blue bars). AlloP served as an additional comparison of anti-inflammatory effects (right red bar) in an independent replication of results from Fig. 2. Furthermore, we confirmed that compounds alone, without LPS-induced inflammation, have no impact on the expression of IL-6 mRNA (Supplemental Figure 1 C). The pattern in Fig. 3 indicates a potential mild correlation between the anti-inflammatory effects and functional impacts on GABA_A_ receptors. The positive modulators *ent*-Etio and AlloP had the most robust anti-inflammatory effects, and negative allosteric modulators of GABA_A_ receptors had least effect.

### 3.3. Structure-activity profile of side-chain oxysterols on LPS-induced inflammation

Oxysterols are oxidized forms of cholesterols that play an important role in brain cholesterol homeostasis, neurodevelopment, and cell signaling [41,42]. Further, some assays have revealed complex effects of oxysterols on inflammation [43,44] and ionotropic glutamate receptors [30]. Thus, we broadened our scope and selected two of the most important groups: 24S-hydroxycholesterol (24S-HC) and 25-hydroxycholesterol (25-HC), to test in BV2 cells challenged with LPS. Our studies also included synthetic analogues of 24S-HC (SGE-201 and SGE-301), as well as *ent*-25-HC and the synthetic 25-HC analogue KK-129 [45].

**Fig. 4.**
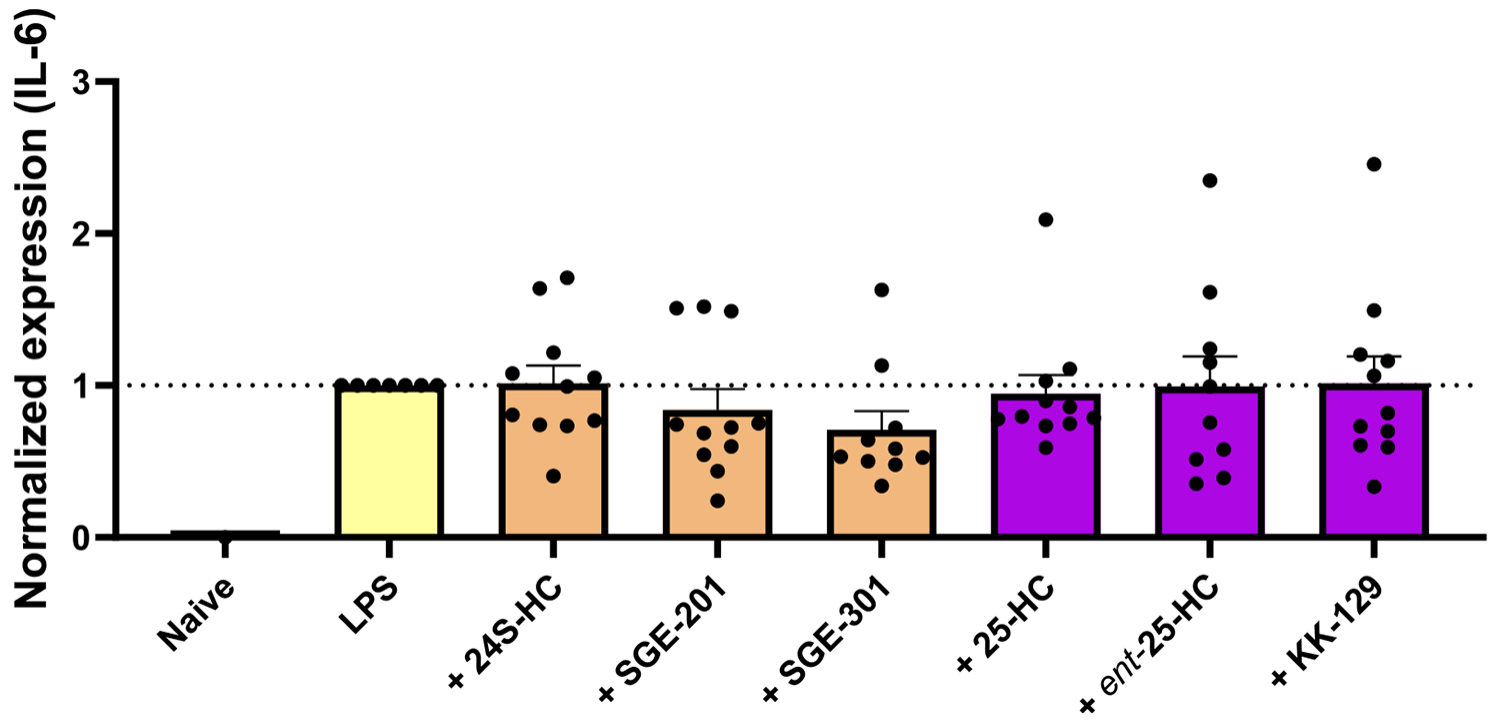
Side-chain oxysterols do not exhibit anti-inflammation effects. A group of 24S-hydroxycholesterol and derivatives (SGE-201 and SGE-301) (light orange bars) and a group of 25-hydroxycholesterol variants (purple bars) were tested. Both groups lacked anti-inflammatory effects in BV2 cells challenged with LPS (yellow bar). The compounds were used at a concentration of 10 μM. Statistical analyses were performed by the ordinary one-way ANOVA test, and the *P* values are indicated above the bars. The analysis revealed no difference between the LPS treated group and the co-treatment groups.

Fig. 4 shows that 24S-HC, along with SGE-201 and SGE-301 did not counteract the elevated mRNA expression of IL-6 induced by LPS administration (orange bars). Similarly, 25-HC and *ent*-25-HC, along with the analogue KK-129, exhibited no impact on the transcriptional changes of IL-6 (Fig. 4 purple bars). Furthermore, oxysterol analogues alone, without LPS-induced inflammation, did not alter IL-6 mRNA expression (Supplemental Figure 1D).

### 3.4. Viability evaluation

An alternative explanation for the effect of neuroactive steroids is that they negatively impact cell survival in the presence of LPS to decrease cytokine levels. To test this possibility, we chose a set of compounds that elicited decreases in cytokine levels and repeated treatments, followed by Trypan Blue staining. After overnight treatment with LPS or LPS plus steroid, cell morphology and Trypan Blue uptake were examined by light microscopy. Quantitative evaluation of cell viability revealed no significant difference between the groups (Supplemental Figure 3 A & 3B). We also tested the possibility that compounds alone have impact on the cell viability (Supplemental Figure 3 C) and found no effect. Overall, we conclude that changes to cell survival cannot explain the decreased pro-inflammatory cytokine expression in the presence of neuroactive steroids.

### 3.5. Correlation of inflammatory cytokine IL-6 protein in BV2 cell culture medium with its transcription

**Fig. 5.**
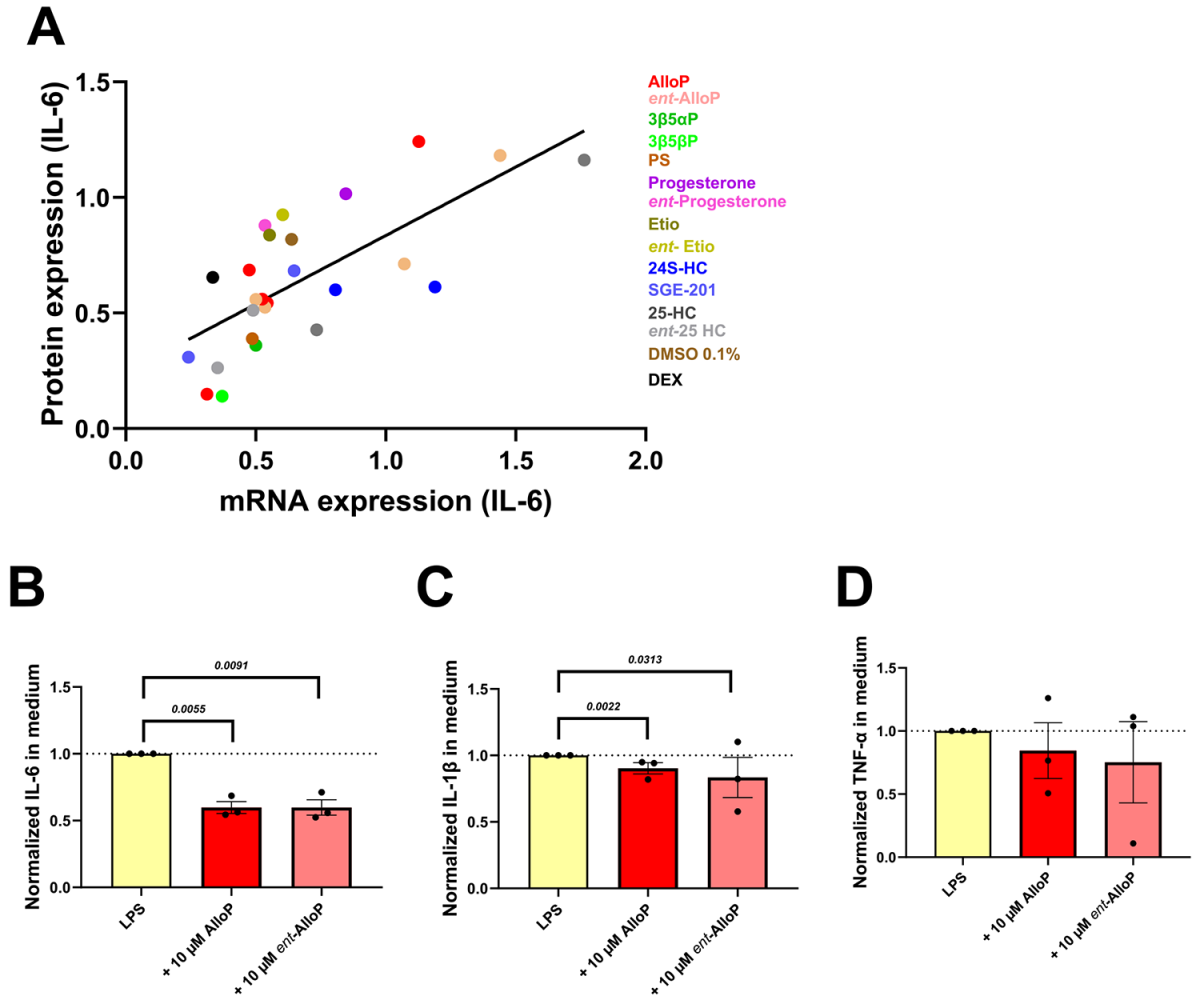
Correlation of inflammatory cytokine (IL-6) protein abundance in BV2 cell culture medium and its mRNA expression in cell lysates. A. Luminex cytokine panels were used to measure the protein expression of inflammatory cytokines in the microglial BV2 cell culture medium (n = 6 plating), compared with matched to BV2 a real-time qPCR assay. IL-6 is illustrated as a robust representative cytokine. The solid line represents linear corresponding between the protein abundance and the mRNA abundance. Sample identities and datasets are illustrated with different colors. Simple linear regeneration analysis, Pearson *r* = 0.72, *R^2^* 0.52, *F (_1,24_)* = 25.87, *P* < 0.0001. B-D. IL-6, IL-1β and TNF-α protein levels from the multiplex experiment. Cytokine levels were normalized to the LPS control within each plating. Statistical analyses were performed using the Wilcoxon signed-rank test, and the corresponding *P* values are displayed above the bars.

Finally, to ensure that transcription assays in Figs. 2-4 reflect protein expression in the experiments, we evaluated the correspondence between transcriptional results and protein expression of pro-inflammatory cytokines found in BV2 culture medium. We used a predesigned MilliPlex of 32 mouse cytokines to measure the contents in the culture medium. The samples selected for protein analysis were matched with their real-time qPCR assay samples (n = 6 platings). We focused in IL-6 as the most reliable pro-inflammatory transcriptional marker (Fig. 2). Fig. 5 exhibits a strong linear correlation between cytokine IL-6 protein production and their mRNA expression, indicating that the transcriptional changes observed are reflected at the protein level. Fig. 5B-D shows IL-6 and IL1-β are significant toward an effect of AlloP and *ent*-AlloP on other pro-inflammatory cytokines as well, paralleling the effect on RNA levels in earlier figures.

## 4. Discussion

Our study provides evidence that anti-inflammatory effects can be dissociated from effects on ionotropic receptors of steroid and sterol neuromodulators. The neurosteroid AlloP and *ent*-AlloP exert anti-inflammatory effects against LPS-induced inflammation in murine BV2 microglial cells, but only e*nt*-AlloP lacks strong GABA_A_ receptor effects [31]. Another entiomeric pair, Etio and *ent*-Etio also exhibited anti-inflammatory effects. In this case, the natural enantiomer exhibits weak GABA_A_ receptor activity [46]. Several other steroid and oxysterol-like structures failed to affect inflammatory markers in this cellular assay. Our observations support other recent work demonstrating anti-inflammatory effects of neuroactive steroids [47-49]. Our work further reveals that another enantiomeric pair (Etio and *ent*-Etio) has similar anti-inflammatory effects, while some GABA_A_ receptor neutral neurosteroid analogues appear to have limited effects. Overall, the results broaden our understanding of neuroactive steroid effects beyond their roles in receptor modulation and point to selective mechanistic effects.

Inflammation is likely a shared etiological risk factor in major depressive disorder and other psychiatric and metabolic disorders [9, 10]. The relationship between inflammation and depression is complex, with inflammation both potentially initiating and perpetuating depressive symptoms. An LPS-induced depression-like model in mice is commonly used to study the mechanisms of inflammation-associated depression and the therapeutic effects of drugs [13,44]. We used BV2 microglia cells to mimic the cellular immune response to LPS as a tool for rapid drug screening. Consistent with others [13,50], we observed a robust increase in cytokines, including IL-1β, IL-6 and TNF-α, with transcriptional expression closely matched by protein expression for IL-6. We observed robust transcriptional effects on IL-1β and IL-6; the weaker effects on TNF-α transcription (Fig. 2) might be explained by different time courses of expression for the different cytokines [51]. In addition, anti-inflammatory cytokines remain to be evaluated in future experiments that employ a more prolonged time course design. Typically, anti-inflammatory cytokine expression increases later than pro-inflammatory cytokines [52]. We did not observe increases in IL-10 protein expression in our multiplex analysis at the time point evaluated (https://doi.org/10.6084/m9.figshare.30053995.v1).

AlloP has pleiotropic actions in the CNS [53,54]. These include strong potentiation of GABA_A_ receptor function [22,55,56], inhibition of the hypothalamic-pituitary-adrenal axis [57] as well as inhibition of pro-inflammatory and neuroimmune activation in macrophages and brain [48,49]. Further, our group also suggested that neuroactive steroids, including AlloP and *ent*-AlloP, may modulate autophagy in a cell-specific or context-specific manner [58-60]. This body of evidence highlights the multifaceted roles of neuroactive steroids in both physiological and pathological conditions and could be a useful guide to develop strategies for studying the effects of other neurosteroid analogues on inflammatory signaling.

Brexanolone (AlloP) was approved by FDA as the first antidepressant specifically for postpartum depression (PPD) [16-18]. It appears to inhibit inflammatory signaling in PPD patients within six hours of infusion, with these effects correlating with its therapeutic efficacy as measured by reduction in depression scores [23]. Zuranolone, an orally active neurosteroid analogue, was also approved for the treatment of PPD [61]. While the cellular mechanisms of the durable therapeutic effects of these drugs remain unclear, our investigation suggests that AlloP could work partly through anti-inflammatory effects. Furthermore, we show that *ent*-AlloP has a similar impact on cytokine suppression, identifying it as a promising candidate for therapeutic development. Because of its lack of impact on GABA_A_ receptors, *ent*-AlloP may offer fewer side effects, particularly sedation.

Cholesterol, the parent molecule for endogenous neurosteroid synthesis, plays a crucial role in the brain with the majority of its metabolism in neurons via CYP46A1 to produce the oxysterol 24S-HC [62]. Unlike cholesterol, 24S-HC can cross the blood-brain barrier (BBB), making it a mechanism for diminishing excess cholesterol in the brain and a potential biomarker for neurodegenerative diseases such as Alzheimer’s disease (AD) and Parkinson’s disease (PD) [61,63,64]. Another oxysterol, 25-HC is produced by macrophages and microglia and amplifies inflammatory signaling [27-29,65-67]. In other contexts, 25-HC modulates and mitigates inflammation [66]. In the current investigation, exogenous 25-HC and variants had no discernible impact on cytokine expression. This may be the result of the simplified culture system and experimental design of our screen, which used a single LPS time point and concentration. Different conditions could alter the results in our assay, raising the importance of additional studies under more complex conditions.

The mechanisms underlying the anti-inflammatory effects of neuroactive steroids in LPS-induced inflammation remain unclear. Previous studies have shown that endogenous AlloP inhibits the activation of inflammatory toll-like receptor 4 (TLR4) signaling in Raw264.7 macrophages and the brains of selectively bred alcohol-preferring (P) rats [49]. Additionally, AlloP enhances the production of the anti-inflammatory mediator cytokine IL-10 via the endosomal TRIF-dependent TLR4 signaling pathway in male but not female P rats [68]. Further, under LPS-induced inflammation, 25-HC has been shown to impair hippocampal plasticity [44,47] contributing to the effects of toll-like receptor activation. These findings, together with our own, highlight the intricate relationships between inflammation, immunity, alcohol addiction, and learning and memory. Further research is needed to elucidate the precise mechanisms and targeted pathways involved.

In conclusion, our findings expand the understanding of structureactivity relationships for neuroactive steroids and oxysterols with respect to anti-inflammatory properties. This knowledge could guide the development of novel pharmaceuticals for targeting neuroinflammatory-related neuropsychiatric disorders in the absence of effects on ionotropic receptors.

## Supplementary Material

1

## Data Availability

Data will be made available on request.

## Appendix A. Supporting information

Supplementary data associated with this article can be found in the online version at doi:10.1016/j.jsbmb.2026.106969.

## Abbreviations:

AlloP: allopregnanolone
ent-AlloP: ent-allopregnanolone
ent: enantiomer
24S-HC: 24-S-hydroxycholesterol
25-HC: 25-hydroxycholesterol
Etio: etiocholanolone
PS: pregnenolone sulfate
LPS: lipopolysaccharide
DEX: dexamethasone
IL-1β: interleukin 1β
IL-6: interleukin 6
TNF-α: tumor necrosis factor α
MRNA: messenger ribonucleic acid
QPCR: quantitative polymerase chain reaction
TLR: toll-like receptor
