# Supplementary material for: Structure-activity relationships of steroid and sterol neuromodulators on inflammatory markers in a murine microglial cell line
Source: J Steroid Biochem Mol Biol. Author manuscript; Available in PMC 2026 May 14. (PMC13175375; doi:10.1016/j.jsbmb.2026.106969)
Supplement: 1 [file NIHMS2163867-supplement-1.docx]

**Supplemental Figure 1. Lack of effects of solvent and neuroactive steroids alone on cytokine expression without LPS co-application in BV2 cells. A.** DMSO (0.1%) solvent did not affect IL-6 transcription in the presence of LPS (N=8 separate platings). Statistical analysis was performed using the Mann-Whitney test, with the *P* value indicated above the bars. **B.** The mRNA expression of cytokines was investigated using a real-time quantitative PCR assay. Neither IL-1β, IL-6 nor TNF-α was affected by neuroactive steroids in the absence of LPS-induced inflammation. For comparison, cytokine expression levels following LPS administration (yellow bars) are displayed. **C.** Other Neuroactive steroids used in the experiments showed no significant effects on IL-6 transcript levels when LPS was absent. **D.** Oxysterols and their variants alone did not affect IL-6 transcript levels. Data represents N=3-4 separate plating.

**Supplemental Figure 2. Evaluation of BV2 cell viability following treatment with neuroactive steroids under LPS-induced inflammation.** BV2 cells were challenged with LPS overnight and co-treated with neuroactive steroids. Cell viability was assessed by Trypan Blue staining. A subset of neuroactive steroids that affect cytokine transcription were tested at 10 µM. **A**. Representative images from the experimental groups. In these grayscale images, black dots denote Trypan Blue-positive cells, indicating loss of membrane integrity. Scale bar = 50 µm. **B.** Viability of cells following LPS treatment and co-treatment with select neuroactive steroids was compared with a naïve group. Statistical analyses were performed using Wilcoxon signed-rank test. No differences in cell viability were detected. P values above the bars indicate comparisons to the naïve group. Data represent N = 4 independent experiments. **C**. Viability was evaluated for neuroactive steroids in the absence of LPS. *P* values above the bars indicate comparison with the naïve group. Treatment with compounds alone did not detectably alter cell viability compared to the naïve group in 3 independent experiments.

| **Compound** | **GABA effect** | **Reference** |
| --- | --- | --- |
| AlloP | Positive | [21,22,54,55] |
| *ent*-AlloP | Positive | [31,32] |
| Progesterone | Neutral | [38] |
| *ent*-progesterone | Neutral | 1.12 ± 0.10 (n = 4) |
| Etio | Neutral | [38,39,45] |
| *ent*-Etio | Positive | [38,39,45] |
| Pregnenolone sulfate | Negative | [32] |
| 3β5αP | Negative | [32] |
| 3β5βP | Negative | [32] |

**Supplemental Table. Structure-activity profile of neuroactive steroids on** **GABA****_A_ receptors.** The effects of neurosteroids on GABA_A_ receptors were categorized as **positive**, **neutral**, or **negative**. References supporting reported GABA_A_ receptor actions are provided. *ent*‑progesterone has not been previously published; the activity value reported here is from screening on cultured hippocampal neurons obtained at 10 µM *ent*‑progesterone by procedures standard in our publications [71].

**Acknowledgement**

The authors thank Taylor Family Institute for Innovative Psychiatric Research for supporting this project. This work was supported by NIMH grants RO1 MH123748 (SM), P50 MH122379 (SM and CFZ) and R01MH110550 (DFC).

**Declaration of Competing Interest**

CFZ previously served on the Scientific Advisory Board of Sage Therapeutics and held equity in the company. Sage Therapeutics was not involved in this work. The other authors declare that they have no known competing financial interests or personal relationships that could have appeared to influence the work reported in this paper.

**Reference**

[71] M. Qian, Y. Xu, D.F. Covey, Synthetic routes to trifluoromethylphenyl diazirine photolabeling reagents containing an alkyne substituent (TPDYNE) for chemical biology applications, RSC Adv. 13 (2023) 36484–36492, doi:10.1039/d3ra07260f.
